# Perioperative Stroke in a Patient Undergoing Noncardiac, Non-Neurosurgical Procedure: A Case Report

**DOI:** 10.7759/cureus.9570

**Published:** 2020-08-05

**Authors:** Lakshmi N Kurnutala, Suwarna Anand

**Affiliations:** 1 Anesthesiology, University of Mississippi Medical Center, Jackson, USA

**Keywords:** anesthesia, stroke, perioperative period, noncardiac surgery, nonneurosurgical procedure

## Abstract

Perioperative stroke is a focal or global neurological deficit lasting more than 24 hours, which occurs during the surgery or within 30 days following surgery. Medications administered during anesthesia mask the symptoms of stroke in the perioperative period and make the early diagnosis of stroke difficult. Postoperative endothelial dysfunction and surgery-induced hypercoagulable state are some of the factors contributing to perioperative stroke. This report describes a case of perioperative stroke in a patient with an unremarkable intraoperative course following otolaryngology surgery. Vigilance, early diagnosis, and prompt treatment with the help of the acute stroke team are pivotal in improving patient outcomes.

## Introduction

Stroke is a disastrous complication of surgery and an important cause of morbidity and mortality, particularly in geriatric patients. It presents a significant burden to the healthcare system and leads to impaired quality of life for patients. The mortality rate following a stroke due to general surgery is estimated at 26% and increases to 87% in patients who have had a previous stroke [1]. The incidence of stroke is approximately 0.1-1.9% in noncardiac, non-neurologic, and non-major surgeries [2] and estimated to be as high as 10% in patients undergoing cardiac or neurosurgery [3]. The majority of the perioperative strokes occur postoperatively within seven days (rather than intraoperatively) and are ischemic rather than hemorrhagic [4]. Among ischemic strokes, thrombosis is the most common cause in general surgery as opposed to cardiac surgery, which is associated with embolic stroke. In this paper, we discuss our experience with perioperative stroke in a patient who had undergone otolaryngology surgery.

## Case presentation

**Figure 1:**
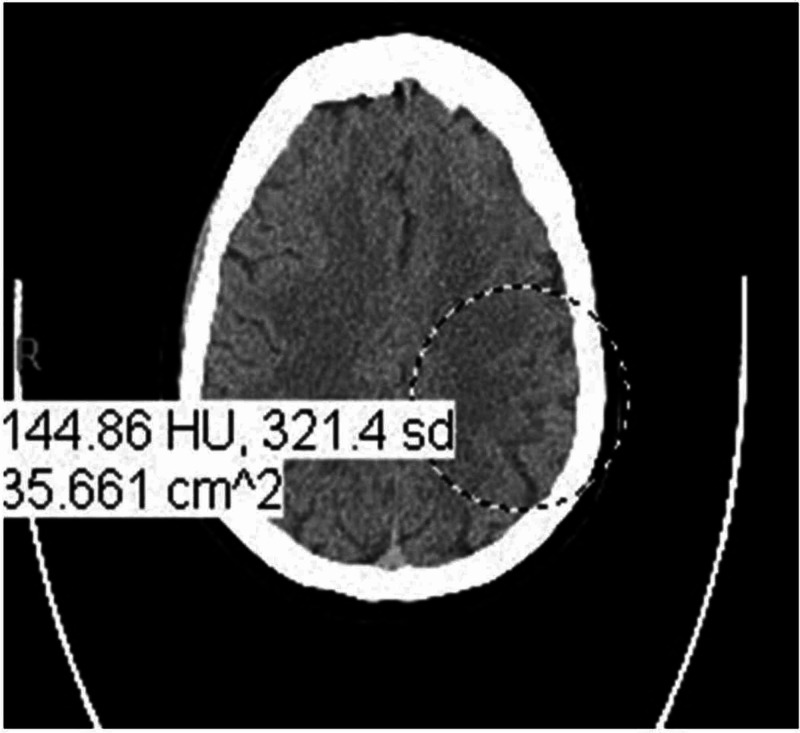
Non-contrast CT of the brain The image shows evidence of early left middle cerebral artery territory infarct CT: computed tomography

A 62-year-old Caucasian female patient with a history of diabetes, migraine, and hyperlipidemia [American Society of Anesthesiologists (ASA) physical status 2] [5] underwent direct laryngoscopy and vocal cord injection for unilateral vocal cord paralysis with dysphonia under general anesthesia. The patient had experienced a delayed emergence from anesthesia a few years ago from a similar surgery. Preoperative labs had been within normal limits and EKG showed normal sinus rhythm (NSR). The patient was admitted to the post-anesthesia care unit (PACU) following an unremarkable general anesthesia course with stable intraoperative vitals after a 30-minute surgical time. She extubated in the operating room after meeting the extubation criteria. In the PACU, the patient appeared sedated for an extended period with the absence of spontaneous movement of her extremities and lack of response to verbal commands except for spontaneous eye-opening. The patient had received 2 mg of midazolam and 100 mcg of fentanyl intraoperatively; no other medications were administered in the PACU. She was treated with naloxone and flumazenil to exclude opioid and benzodiazepine overdose respectively. Her PACU vitals were stable, and arterial blood gas analysis showed blood sugar of 231 mg/dl with normal electrolytes. Two hours after her arrival to PACU, when the patient was more awake, she was found to have a flat affect, right-sided weakness, and right-sided facial droop. The acute stroke team was consulted and, after a quick neurological assessment, the patient was taken for an emergent CT scan of the brain. A non-contrast CT of the brain showed evidence of early left middle cerebral artery (MCA) territory infarct, and her calculated National Institute of Health Stroke Score (NIHSS) was 26 (Figure 1) [6].

As her surgical procedure was minor, she was treated with tissue plasminogen activator (tPA) and was admitted to the neurosurgical intensive care unit for monitoring (three hours postoperatively and one hour after activating code gray). Due to the midline shift, the patient was started on hypertonic saline therapy. Hypertension and blood glucose were kept under good control. Further diagnostic tests were performed to evaluate the cause of the stroke. EKG throughout the course showed NSR. CT angiogram of head and neck showed scattered atherosclerosis without evidence of hemodynamically significant stenosis of major intracranial and cervical vessels. Transthoracic echocardiography (TTE) with contrast performed the next day showed no evidence of thrombus with a good left ventricular (LV) and right ventricular (RV) function, no intracardiac shunt, and no valvular abnormality. The patient was started on aspirin 81 mg, and she underwent aggressive rehabilitation therapy to improve her functional status. She was discharged after 22 days of in-hospital stay to a nursing home for chronic rehabilitation therapy.

## Discussion

The incidence of perioperative stroke in patients undergoing noncardiac, non-neurosurgical procedures is approximately one in 1,000, but it increases the mortality by eightfold [7]. Medications given during anesthesia (general anesthesia, sedation, and regional anesthesia) mask the symptoms of stroke, thereby making the diagnosis of stroke difficult during the perioperative period [1]. No screening tool to detect the incidence of perioperative stroke in patients undergoing anesthesia can be found in the current literature.

Preoperative strategies to mitigate the risk of stroke

Timing of Elective Surgery After an Acute Stroke

Cerebral autoregulation is impaired following acute stroke, making the brain more vulnerable to hypotension. Patients with prior stroke are at increased risk of subsequent stroke for nine months following the stroke (Table 1) [2].

**Table 1:** Risk factors for stroke [1,2]

Perioperative risk factors for stroke	Independent risk factors for stroke
Advanced age	Atrial fibrillation
Previous history of stroke	Renal disease
Type of surgery	Hypertension
Pain	Diabetes
Hypercoagulable state	Smoking
Unstable hemodynamics	Vascular disease
Blood loss	Congestive heart failure

Perioperative management with anticoagulants and anti-platelet drugs is based on the delicate balance between the risks of excessive perioperative bleeding vs. the rebound hypercoagulability associated with the prothrombotic state of surgery. Perioperative contributing factors like postoperative endothelial dysfunction leading to plaque rupture, reactive vasospasm, and thrombus formation may predispose patients to thrombotic risk. Withholding antiplatelet agents or anticoagulants in the perioperative period may aggravate surgery-induced hypercoagulability and increase the risk of stroke; 14% of strokes after general surgery are associated with atrial fibrillation, highlighting the importance of embolism and the hypercoagulable state [8-10]. The rate of thromboembolism is low in both bridged and non-bridged patients [11,12]; the decision to initiate anticoagulation post-surgery is based on the type of surgery, adequate hemostasis, and individual risks factors. As per the Perioperative Ischemic Evaluation (POISE)-2 trial, perioperative aspirin was associated with a reduced risk of stroke but was linked with major bleeding, though this benefit was not seen in patients who had already been on aspirin therapy [13]. Statin therapy is usually continued in the perioperative setting in patients with acute stroke or initiated two weeks prior to surgery to reduce the risk of neurological deterioration or death [1]. Statins confer their protection through their anti-inflammatory effect [1].

Intraoperative considerations

POISE-1 trial demonstrated that perioperative metoprolol (beta-blocker) in noncardiac surgical patients increased the risk of perioperative stroke [14]. Mashour et al. found a 3.3-fold unadjusted increased risk of perioperative stroke in these patients, possibly due to impaired cerebral tissue oxygen delivery via decreased cerebral vasodilation [15]. Angiotensin-converting enzyme inhibitors in the perioperative setting cause hypotension, which is unresponsive to conventional therapy, thereby increasing the risk of stroke. As there is no blood pressure threshold for stroke, there is no consensus on perioperative blood pressure targets. It is reasonable in patients at high risk for perioperative strokes to maintain mean or systolic blood pressures within 20% of preoperative blood pressure [16,17]. In patients at high risk for stroke, it is recommended to keep the perioperative glucose less than 180 mg/dl to improve neurological outcome following cerebral ischemia [18].

Postoperative considerations

Perioperative stroke has a negative impact on recovery from surgery, as observed in our patient. Postoperative diagnosis of stroke and time of onset can be challenging as residual anesthetic effects may mask neurologic deficits from a stroke. A detailed clinical evaluation including neurological examination using the NIHSS, blood pressure, oxygen saturation, temperature, EKG, basic metabolic panel, complete blood count, and coagulation status should be performed. Non-contrast CT of the brain is used to promptly diagnose and differentiate the type of stroke and to initiate immediate therapy.

Treatment

Ischemic stroke is the most common perioperative stroke, and its treatment involves a multidisciplinary approach consisting of anesthesiology, neurology, interventional neuroradiology, and primary surgical service. Treatment with recombinant tPA (intravenous thrombolysis) depends on the type, location, severity of the stroke, and the type of surgical intervention the patient has undergone. A history of major surgery within the past 14 days is an exclusion criterion for the administration of tPA because of the risk of surgical-site bleeding [19]. Endovascular thrombectomy (EVT) in patients with large vessel occlusion has led to effective recanalization and a better clinical outcome, without the additional risk of hemorrhagic complication [20]. However, the functional outcome and mortality rate were significantly worse in patients with perioperative stroke. Patients with acute ischemic stroke should have a cardiac assessment performed with troponin and EKG for the initial 24 hours as myocardial infarction and cardiac arrhythmias are likely. Postoperative conditions like pain, stress, nausea, vomiting, hypovolemia or hypervolemia, and hypoxia should be prevented or treated to improve perioperative stroke outcome. Systolic blood pressure above 180 mmHg and diastolic blood pressure above 105 mmHg should be promptly treated with anti-hypertensives [1,2]. Arterial saturation should be maintained above 94%, and patients with depressed consciousness and bulbar dysfunction should be mechanically ventilated for airway support and respiratory compromise.

## Conclusions

Perioperative stroke is a debilitating complication in noncardiac and non-neurological surgery. Perioperative risk-factor modification, early diagnosis involving physical examination, the NIHSS, and CT imaging of the brain combined with a multidisciplinary team approach toward treatment generally improve neurological outcomes in this patient population.

## References

[1] Ng JL, Chan MT, Gelb AW (2011). Perioperative stroke in noncardiac, nonneurosurgical surgery. Anesthesiology.

[2] Bateman BT, Schumacher HC, Wang S, Shaefi S, Berman MF (2009). Perioperative acute ischemic stroke in noncardiac and nonvascular surgery: incidence, risk factors, and outcomes. Anesthesiology.

[3] Selim M (2007). Perioperative stroke. N Engl J Med.

[4] Macellari F, Paciaroni M, Agnelli G, Caso V (2012). Perioperative stroke risk in nonvascular surgery. Cerebrovasc Dis.

[5] Hurwitz EE, Simon M, Vinta SR, Zehm CF, Shabot SM, Minhajuddin A, Abouleish AE (2017). Adding examples to the ASA-Physical Status Classification improves correct assignment to patients. Anesthesiology.

[6] Sato S, Toyoda K, Uehara T (2008). Baseline NIH Stroke Scale Score predicting outcome in anterior and posterior circulation strokes. Neurology.

[7] Vlisides P, Mashour GA (2016). Perioperative stroke. Can J Anaesth.

[8] Hart R, Hindman B (1982). Mechanisms of perioperative cerebral infarction. Stroke.

[9] Larsen SF, Zaric D, Boysen G (1988). Postoperative cerebrovascular accidents in general surgery. Acta Anaesthesiol Scand.

[10] Landercasper J, Merz BJ, Cogbill TH, Strutt PJ, Cochrane RH, Olson RA, Hutter RD (1990). Perioperative stroke risk in 173 consecutive patients with a past history of stroke. Arch Surg.

[11] Douketis JD, Spyropoulos AC, Kaatz S (2015). Perioperative bridging anticoagulation in patients with atrial fibrillation. N Engl J Med.

[12] Steinberg BA, Peterson ED, Kim S (2015). Use and outcomes associated with bridging during anticoagulation interruptions in patients with atrial fibrillation: findings from the Outcomes Registry for Better Informed Treatment of Atrial Fibrillation (ORBIT-AF). Circulation.

[13] Devereaux PJ, Mrkobrada M, Sessler DI (2014). Aspirin in patients undergoing noncardiac surgery. N Engl J Med.

[14] POISE Study Group, Devereaux PJ, Yang H (2008). Effects of extended-release metoprolol succinate in patients undergoing non-cardiac surgery (POISE trial): a randomised controlled trial. Lancet.

[15] Mashour GA, Sharifpour M, Freundlich RE (2013). Perioperative metoprolol and risk of stroke after noncardiac surgery. Anesthesiology.

[16] Bijker JB, van Klei WA, Kappen TH, van Wolfswinkel L, Moons KG, Kalkman CJ (2007). Incidence of intraoperative hypotension as a function of the chosen definition: literature definitions applied to a retrospective cohort using automated data collection. Anesthesiology.

[17] Howell SJ, Sear JW, Foëx P (2004). Hypertension, hypertensive heart disease and perioperative cardiac risk. Br J Anaesth.

[18] Atkins JH, Smith DS (2009). A review of perioperative glucose control in the neurosurgical population. J Diabetes Sci Technol.

[19] National Institute of Neurological Disorders and Stroke rt-PA Stroke Study Group (1995). Tissue plasminogen activator for acute ischemic stroke. N Engl J Med.

[20] Hong KS, Ko SB, Lee JS, Yu KH, Rha JH (2015). Endovascular recanalization therapy in acute ischemic stroke: updated meta-analysis of randomized controlled trials. J Stroke.

